# The emergence of maternal health as a political priority in Madhya Pradesh, India: a qualitative study

**DOI:** 10.1186/1471-2393-13-181

**Published:** 2013-09-30

**Authors:** Tej Ram Jat, Prakash Ramchandra Deo, Isabel Goicolea, Anna-Karin Hurtig, Miguel San Sebastian

**Affiliations:** 1 United Nations Population Fund, UN Office, 41-42, Polytechnic Colony, Shyamla Hills, Bhopal, Madhya Pradesh 462013, India; 2 Department of Public Health and Clinical Medicine, Epidemiology and Global Health, Umeå University, Umeå, SE 90187, Sweden; 3 Swedish Research School for Global Health, Umeå University, Umeå, SE 90187, Sweden; 4 Umeå Centre for Gender Studies, Umeå University, Umeå, SE 90187, Sweden

**Keywords:** Maternal health, Maternal mortality, Political priority, Madhya Pradesh, India

## Abstract

**Background:**

Politics plays a critical role in agenda setting in health affairs; therefore, understanding the priorities of the political agenda in health is very important. The political priority for safe motherhood has been investigated at the national level in different countries. The objective of this study was to explore why and how maternal health became a political priority at sub-national level in the state of Madhya Pradesh in India.

**Methods:**

This study followed a qualitative design. Data were collected by carrying out interviews and review of documents. Semi-structured interviews were carried out with twenty respondents from four stakeholder groups: government officials, development partners, civil society and academics. Data analysis was performed using thematic analysis. The analysis was guided by Kingdon’s multiple streams model.

**Results:**

The emergence of maternal health as a political priority in Madhya Pradesh was the result of convergence in the developments in different streams: the development of problem definition, policy generation and political change. The factors which influenced this process were: emerging evidence of the high magnitude of maternal mortality, civil society’s positioning of maternal mortality as a human rights violation, increasing media coverage, supportive policy environment and launch of the National Rural Health Mission (NRHM), the availability of effective policy solutions, India’s aspiration of global leadership, international influence, maternal mortality becoming a hot debate topic and political transition at the national and state levels. Most of these factors first became important at national level which then cascaded to the state level. Currently, there is a supportive policy environment in the state for maternal health backed by greater political will and increased resources. However, malnutrition and population stabilization are the competing priorities which may push maternal health off the agenda.

**Conclusions:**

The influence of the events and factors evolving from international and national levels significantly contributed to the development of maternal health as a priority in Madhya Pradesh. This led to several opportunities in terms of policies, guidelines and programmes for improving maternal health. These efforts were successful to some extent in improving maternal health in the state but several implementation challenges still require special attention.

## Background

There have been several significant achievements in global health over the past five decades but reducing maternal mortality in the majority of low and middle income countries is still posing a serious challenge. The fifth Millennium Development Goal (MDG) aims at reducing the maternal mortality ratio (MMR) by three quarters by 2015 (from a starting point in 1990) [1]. Recently updated statistics showed that there were an estimated 358,000 maternal deaths in the world in 2008, of which 99% occurred in low and middle income countries. MMR in these regions in 2008 was 290 maternal deaths per 100,000 live births, which represented a 34% decline since 1990. The average annual decline in the global MMR has been 2.3%, which is considerably lower than the 5.5% annual decline necessary to achieve the MDG target [2]. India has achieved an appreciable decline in the MMR from 892 maternal deaths per 100,000 live births in 1972–76 to 212 during 2007–09 [3,4]. Despite this remarkable decline, the current level of MMR in India is still unacceptably high.

It is widely acknowledged that political will plays a crucial role in agenda-setting and the success or failure of any intervention [5,6]. Any issue becomes political priority as a result of many complex processes. Different health issues and initiatives vary in the extent to which they are prioritized by political leaders and policy makers. Political priority is defined as the degree to which political leaders actively pay attention to an issue, the political systems lead to policies and programmes that address the problem and these programmes are supported by financial, technical and human resources [7,8]. Since politics plays a critical role in health affairs, it becomes very important to understand the priorities of the political agenda in health and the factors associated with it.

There have been efforts to assess the political prioritization of safe motherhood at the national level in different countries [8-13]. A study conducted at the national level in India reported that maternal health has been one of the components in the national programme since independence but it has not gained much prominence for several decades. It revealed that maternal health emerged as a political priority in India in 2005 as a result of a confluence of events concerning problem definition, the generation of policy alternatives and political change. The study also highlighted a challenge in terms of making maternal health a political priority at the sub-national level, particularly in the northern states where the majority of India’s maternal deaths occur [12]. In the Indian federal system, the sub-national level is particularly important because health is a state subject under the distribution of powers between the central and the state governments and states play a strong role in the provision of health services.

This study was conducted in Madhya Pradesh, one of the central states in India. Maternal health became a political priority in Madhya Pradesh in 2005 following the emergence of maternal health as a political priority at the national level [14,15]. Prior to this, maternal health and maternal mortality reduction had received some policy attention since it was included in the state population policy of 2000 [16]. However, it could not be institutionalized as a priority backed by the required political and resource support. Currently, the problems of poor maternal health and high MMR have been recognized to a large extent by politicians and policy makers and are appearing prominently on the policy and political agenda in the state. Maternal health has been included in the chief minister’s 100 days priority agenda [15]. It has also been declared a top priority in the state government’s ‘Resolution 2013’ , which guides the policies and programmes of the state for fulfillment of commitments by 2013 [17]. Improving maternal health is also reflected noticeably in the state project implementation plan for the National Rural Health Mission (NRHM) [14]. In order to characterize the processes leading to the emergence of maternal health as a priority at state level it is crucial to study how it happened.

The objective of this study was to explore why and how maternal health became a political priority in Madhya Pradesh.

## Methods

### Theoretical framework

In his multiple streams model, which is particularly useful for examining agenda-setting, John W. Kingdon explains how some subjects rise on agendas while others get neglected [7]. This model has three streams of processes: problems, policies and politics.

The problem stream has three broad elements which help problem generation by influencing politicians and policy makers. These elements are: 1) evidence of the magnitude and severity of the conditions, 2) major events giving public visibility to the conditions and 3) feedback on the implementation of government programmes and polices. The policy stream incorporates the flow of ideas and set of alternatives to address the priority problem. It includes the emergence of new policies and programmes based on policy recommendations. Policy decisions in this stream are influenced by political support or opposition, the credibility of evidences, the views of experts, bargaining by the policy community, active engagement on the part of interest groups, the public acceptability of ideas and the feasibility of the proposals. Finally, developments in the political stream play a significant role in recognizing problems and setting agendas and priorities. These events can range from elections and changes in governments or changes in bureaucracy to movements and advocacy events organized by interest groups. Actors in the political stream are more visible than in the problem or policy streams.

According to Kingdon, the above three streams largely flow independently of one another, flow in parallel and converge at some point in time wherein a window of opportunity for policy changes opens up and the problem becomes a political priority. This leads to policy decisions to address that problem followed by the development of programmes backed with political support and resources. This model has been used previously by researchers in assessing political priorities and analysing agenda-setting in different contexts [12,18-21].

### Study site

Madhya Pradesh is the second largest state in India in terms of geographical area. As per the provisional figures of the 2011 census, Madhya Pradesh has a population of 72,597,565, which constitutes around 6% of the country’s population. With a total area of 301,283 km^2^, 97.74% of the state is rural. Around 73.5% of the population of the state resides in rural areas. The decadal growth rate of the population between 2001 and 2011 was 20.3% which was higher than that of the country (17.64%) during the same period. Population density in the state is 236 persons/km^2^. In 2011, as many as 40% of women in the state were illiterate, whereas the male literacy rate was 80% [22]. The state has 54,903 villages and it is divided into 50 administrative districts. The state has huge geographical, social, economic and cultural variations.

According to the District Level Household and Facility Survey (DLHS-3) 2007–08, 38.8% of mothers were registered in the first trimester of pregnancy, only 7.9% of women received a full antenatal check–up (ANC) during pregnancy, 47.1% deliveries were conducted at health institutions and only 37.1% of women received post-natal care within two weeks of delivery [23].

Madhya Pradesh has been successful in reducing its MMR sharply during the last decade. The MMR of the state in 1998 was 498 [24]. According to the Annual Health Survey (AHS) 2010–11, the recent MMR of the state was 310 [25]. However, at the current level, it remains among the states with the highest MMR in the country. The AHS 2010–11 also reported huge variations between the administrative divisions; MMR in the Gwalior division was 262, whereas in the Shahdol division it was 435 [25]. The state government is aiming to reduce the MMR to 220 maternal deaths per 100,000 live births by the year 2012 [14].

In order to improve maternal health and reduce maternal mortality, the state government is focusing on increasing institutional deliveries, skilled attendance at birth, and strengthening the emergency obstetric care (EmOC) services as main strategies [14]. Efforts have been made to strengthen the planning and monitoring of maternal health interventions, including expanding the availability of infrastructure, human resources, drugs and equipments and their proper distribution so as to meet the needs of underserved areas of the state.

### Study design

This study followed a qualitative design. The data for this study were collected by carrying out interviews with the concerned stakeholders and a review of documents. Semi-structured interviews were carried out with a purposive sample of 20 respondents divided between four stakeholder groups: government officials, development partners, civil society and academics. These groups play a key role in agenda-setting and decision-making on maternal health in Madhya Pradesh. There were 5 respondents in each group. The group ‘government officials’ was defined as state level officials of the health department who have important role in policy making as well as ensuring the implementation of these policies. The respondent group ‘development partners’ included senior officials of international and bilateral development agencies which are supporting the state government on health issues. The respondents in the ‘civil society’ group included the senior representatives of civil society organisations and groups which actively influence the policy debates and play a key role in agenda setting in the state. The respondent group ‘academics’ consisted of senior academicians working in medical colleges and research institutions with significant influence on policy decisions in the state.

The interviews were performed by the first author using an interview guide developed for the study. Interviews were conducted in the capital city and other cities of Madhya Pradesh during 2010. All the interviews lasted between 60 and 90 minutes. The major focus in the study was on the interviews, and data collected from other sources were used to complement the information provided by the interviews.

### Data analysis

All the interviews were carried out in Hindi. The recordings of the interviews were transcribed verbatim and translated into English by the first author. The transcripts were de-identified to ensure anonymity. Analysis of the data was done using thematic analysis [26,27]. All the transcripts were read and re-read several times to gain thorough familiarity with the responses. The ‘OpenCode 3.6’ software was used to manage the process of coding and categorizing the data [28]. The codes from the different transcripts were reviewed while maintaining the principle of constant comparison. Codes containing similar ideas were grouped together. From the grouped codes, broad themes were developed. The framework of Kingdon’s multiple streams model (problem, policy and political) was used to guide the data analysis [7].

### Ethics

Ethical approval for the study proposal was obtained from the Ethics Committee of the Bhopal Regional Technical Centre of the Family Planning Association of India. Informed consent was gained from each respondent after explanation of the study objectives and assurance of the confidentiality of their identity.

## Results

**Figure 1:**
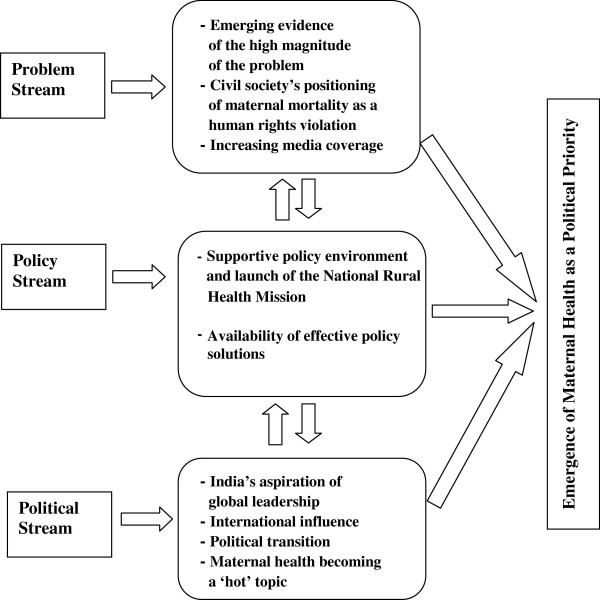
**Emergence of maternal health as a political priority in Madhya Pradesh (Framework- John Kingdon’s Multiple Streams Model).**

The results of this study are presented in the following section using the framework of the public policy agenda-setting process developed by Kingdon (Figure 1). Based on this framework, the factors contributing to the emergence of maternal health as a political priority in Madhya Pradesh are grouped in the three streams: problem, policy and political. The emergence of maternal health on policy agenda was a result of the confluence of these three streams in the year 2005. Relevant quotations from the interviews are provided in italics.

### Problem stream

#### Emerging evidence of the high magnitude of the problem

The respondents stressed that the emergence of credible evidence on the high levels of maternal mortality and low levels of utilization of maternal health care services played a significant role in putting the issue of maternal health on the policy agenda in Madhya Pradesh. Large-scale national demographic surveys, such as National Family Health Survey (NFHS)-1 (1992–93) and NFHS-2 (1998–99), District level Household Survey (DLHS)-1 (1998–99), DLHS-2 (2002 -04) and Sample Registration System (SRS) revealed the issues of poor maternal health and high maternal mortality at national level which also highlighted this issue in Madhya Pradesh. As one of the respondents explained:

“*The problem of maternal mortality became a burning issue* [*at national level and in Madhya Pradesh in the year 2005*] *because different factors such as new information on poor status of maternal health came out from the results of various surveys such as district level household survey* [*2002*–*04*], *the national family health survey and sample registration system* [*1997*–*2003*].” (I-6, civil society representative)

### Civil society’s positioning of maternal mortality as a human rights violation

The respondents acknowledged that following the civil society’s advocacy at the national level for raising the issue of high maternal mortality as a violation of women’s human rights between the year 2000 and 2005, different civil society organizations highlighted this issue in the state as a human rights violation. The organization Human Rights Law Network filed cases in the courts demanding compensation for the victims of maternal deaths who died due to the negligence of the health care system. Jan Swasthya Abhiyan, a network of civil society organizations, organized public hearings in collaboration with the National Human Rights Commission (NHRC) in 2004–05. Based on the findings of these public hearings, the NHRC provided feedback and recommendations to the central and state governments including the government of Madhya Pradesh for strengthening the public health delivery system with a special focus on maternal health services.

A respondent from academics group noted that:

“*Civil society advocacy has also been one of the contributors in the process of highlighting maternal health on policy agenda in Madhya Pradesh*.” (I-16, academic respondent)

A member of Jan Swasthya Abhiyan reported that:

“*We presented* [*in 2004*–*05*] *before the NHRC panel the facts and figures regarding high levels of maternal mortality in the state*, *the poor state of the maternal health care delivery system and very low levels of utilization of maternal health services*. *We also presented the testimonies of women who were denied maternal health services by the government hospitals and case studies of maternal deaths where women died because of the lack of appropriate emergency obstetric care services*.” (I-10, civil society representative)

### Increasing media coverage

Increasing media coverage also played a very significant role in highlighting the issue. The increased media attention mainly started at national level from the year 2000 when maternal mortality reduction was declared as one of the MDGs. It was followed by increased reporting on the issues of poor maternal health and higher magnitude of maternal mortality in the state by electronic and print media. Media continuously reported the poor availability and quality of maternal health services, the denial of maternal health care and cases of maternal deaths, attracting the attention of politicians and policy makers. It was described thus by different respondents:

“*Media played a pivotal role in highlighting these problems and issues* [*since the year 2000 in the country and Madhya Pradesh state*].” (I-20, academic),

“*These problems are getting attention of politicians and policy makers* [*in Madhya Pradesh*] *because of huge numbers of news items in papers and electronic media* [*after the year 2000*]…” (I-11, development partner)

“*Media continuously highlighted the high maternal mortality in the country and Madhya Pradesh state as a human rights issue*. *More media attention on maternal mortality in Madhya Pradesh started from the year 2000 after the millennium declaration as Madhya Pradesh was among the states with highest maternal mortality ratio in the country*” (*I***-***12*, development partner),

“*Some credit can also be given to civil society for their advocacy and lobbying for more attention to maternal health by the government and also the media for highlighting the high levels of maternal mortality on large scale*.” (I-5, government official),

“…. *the media attention that the results of surveys such as NFHS*-*2* [*1998*–*99*, *results published in April 2001*], *DLHS*-*1* [*1998*–*99*, *results published in August 2001*], *DLHS*-*2* [*2002*–*04*] *generated in Madhya Pradesh during the period 2000 to 2005*, *forced the state government to pay attention to the problems of poor maternal health and high maternal mortality ratio and work towards addressing these issues*.” (I-15, development partner)

### Policy stream

#### Supportive policy environment and launch of the National Rural Health Mission

The respondents expressed that the adoption of the state population policy in 2000 initiated the supportive policy environment for maternal health and maternal mortality.

The policy proposed some key interventions for reducing maternal mortality such as increasing registration of pregnant women, providing full range of ANC services to all pregnant women, creating pregnancy testing facilities at each health sub-centre, raising the proportion of institutional deliveries, ensuring that trained birth attendants assist in child births and ensuring medical termination of pregnancy services at health facilities at sub-district level. These policy provisions prepared the ground for larger policy interventions for improving maternal health in the state.

The respondents also perceived that the launch of the NRHM contributed significantly to make maternal health improvement and maternal mortality reduction a priority agenda in the state. Following the launch at national level, the state government started the NRHM in the state in 2005. Madhya Pradesh was selected by the central government among 18 out of 28 states for comprehensive and focused support under the NRHM based on its poor situation in terms of maternal and child health. The goals of the NRHM in Madhya Pradesh were consistent with the MDGs, the Common Minimum Programme of the national government, the state population policy and the International Conference on Population and Development (ICPD) programme of action. The contribution of supportive policy environment and launch of the NRHM in the state was explained by different respondents as follows:

“*The launch of National Rural Health Mission in 2005 pushed the state government for making maternal health a political priority in Madhya Pradesh as it focused on reducing maternal mortality and it also provided additional funding for interventions and services for improving maternal health and reducing maternal mortality*.” (I-17, academic respondent)

“*There is now positive policy and programmatic environment in the state and at the national level for improving maternal health*. *The commitment of government through state population policy 2000 and the state project implementation plans for national rural health mission 2005 and for subsequent years have also accepted the poor state of maternal health and increased attention in both of these documents has been given for improving maternal health*.” (I-6, civil society representative)

### Availability of effective policy solutions

The respondents observed that by the year 2005 several policy solutions, which had been proved to be successful in improving maternal health and maternal mortality in other settings, became available. The availability and effectiveness of these policy solutions encouraged the state government to adopt them.

As noted by different respondents:

“*The availability of interventions and policy alternatives such as emergency obstetric care*, *skilled attendance at birth*, *that could work and reduced maternal mortality sharply in other countries and in Kerala state made us to think that if other countries and states can reduce maternal mortality drastically than why can*’*t we do it*.” (I-4, government official)

“*The state government adopted new policy solutions in the year 2005 for improving maternal health*, *which included improving antenatal care*, *promoting community monitoring of maternal health services*, *increasing institutional deliveries*, *strengthening referral transportation and improving the availability and quality of emergency obstetric care services*.” (I-11, development partner)

Four key strategies were adopted by the state government in 2005 for improving maternal health. First, an improvement in the coverage and quality of ante-natal care services was sought through the mobilization of accredited social health activists (ASHAs). Activities included strengthening the conducting of regular village health and nutrition days (VHNDs) and forming village level committees for community monitoring of health services and improved accountability of the health system. The second strategy was to avoid the three delays (delay in deciding to seek care, delay in reaching the health facility and delay in receiving quality care once at the health facility) through continuing education for pregnant women and family members and strengthening referral transportation with service delivery by improving the health infrastructure in rural areas. Third, the number of institutional deliveries was to be increased through capacity building of health care providers for skilled birth attendance and providing incentives to pregnant women to come to health institutions for delivery. Fourth, the EmOC services were to be strengthened through the identification and upgrading of health institutions as comprehensive EmOC and basic EmOC centres, capacity building of health care providers in EmOC and initiating public-private partnership to ensure institutional delivery services for pregnant women in rural areas where government health institutions were not available.

State, divisional and district level officials regularly monitored the progress of maternal health interventions through review meetings and field visits. The current chief minister also gave special attention to the progress of maternal health programmes in the state.

However, there were several challenges at the implementation level making it difficult to achieve significant results from these efforts. These include the inadequate availability of infrastructure and technical human resources and especially their inequitable distribution in the rural and tribal areas of the state. No significant effort has been made to address the social determinants of maternal health in the state such as poverty, illiteracy and gender-based discrimination.

### Political stream

#### India’s aspiration of global leadership

The respondents expressed the view that India’s economic growth rate had achieved new heights during the last few decades which raised the aspirations of the Indian government for its role of assuming global leadership. However, the social indicators did not improve significantly in comparison with the economic growth during the same period. The benefits of economic growth were not distributed equitably among all sections of society. Realizing the importance of improved levels of social indicators, India has started to pay more attention to improving health, education and other social indicators since 2004. As one of the respondents noted:

“*With very high levels of economic growth*, *India is aspiring to become a global leader but problems such as very high levels of maternal mortality*, *high infant mortality and high malnutrition rates are creating hurdles for India to realize this aspiration because it shows that the distribution of the benefits of high levels of economic growth is not equal among the different sections of society*. *Therefore the Government of India is very serious about addressing these problems and specially the problem of maternal mortality*.” (I-19, academic)

### International influence

The respondents stressed that the declaration of MDGs in 2000 also played a significant role in the emergence of maternal health as a political priority in India and in Madhya Pradesh. Global estimates of maternal mortality levels in 2000, published by UN agencies in 2004 showed that India had the largest number of maternal deaths in the world. This publication pressurized the Government of India to prioritize the issue of maternal health and also compelled the state government in Madhya Pradesh to take the issue of maternal health seriously. As reported by different respondents:

“*The Millennium declaration of MDGs* [*in the year 2000*] *also came and national and state level governments made commitments to achieve these goals*. *MDG five worked as norm setting so that now high maternal mortality will not be accepted and all countries signing the declaration will have to make conscious efforts to improve maternal health and reduce maternal mortality*.” (I-5, government official)

“*Publications of different international agencies i*.*e*. *WHO*, *UNFPA and Unicef between 2000 to 2005 specially the global estimates of maternal mortality created huge awareness about maternal health and maternal mortality among common public*, *media*, *civil society and policy makers in Madhya Pradesh as well as at national level in India*.” (I-14, development partner)

“*Millennium development goals and goals of 11*^*th*^*five year plan are very much in front of us and we have to keep comparing our progress in strengthening maternal health care delivery services and reduction in maternal mortality as our state has been among the states with highest maternal mortality ratio*.” (I-2, government official)

### Political transition

The 2004 parliamentary elections in India played a noteworthy role in the process of the emergence of maternal health as a political priority, with the new government promising to increase public spending on health to 2% to 3% percent of the Gross Domestic Product by the year 2010. This was expressed by one respondent as follows:

“*Political transition at the national level in the 2004 general election and the United Progressive Alliance*’*s coming to power and including maternal mortality reduction as one of the commitments in the Common Minimum Programme also helped in making maternal health a priority at national and state levels*.” (I-5, government official)

In order to realize the promise made in the CMP, the prime minister of India launched the NRHM in April 2005, with the aim of reducing maternal mortality to 100 per 100,000 live births by 2012. The central government started providing additional funds to the state governments under the NRHM and also increased its dialogue with the state governments which motivated the state government in Madhya Pradesh to prioritize the issue of maternal health.

Political transition at the state level also increased the commitment of the state government to improving the status of maternal health. The current chief minister Mr. Shivraj Sing Chauhan, who came to power in 2005, seems to be very serious about improving the status of the social and economic indicators of the state. Some respondents observed that:

“…*at the state level the current chief minister coming into power has brought social sector development issues including improving maternal health*, *onto the policy agenda*.” (I-7, civil society representative)

“*The current chief minister of the state has declared maternal health as an important political agenda in his 100 days programme and constituted a special advisory cell to monitor progress and to give regular feedback*.” (I-11, development partner)

### Maternal health becoming a ‘hot’ topic

Increased debates and discussions on maternal health helped sensitize the politicians on the issues related to maternal health. An analysis of question and answers in the state’s legislative assembly sessions in 2005 revealed that 71 questions related to maternal health and health infrastructure in the state were asked in the assembly by legislators from both- the ruling party and the opposition, in comparison with only 13 questions in 2000. The increased questioning from the legislators also forced the state government to make the issue of maternal health a priority. As noted by one respondent:

“*The issues of the poor status of maternal health services and unacceptably high levels of maternal mortality started being discussed and debated in political and social forums which played a significant role in making these problems burning issues*. *The number of questions on maternal health asked in the state legislative assembly also increased and that attracted the attention of policy makers*.” (I-11, development partner)

## Discussion

The status of maternal health in Madhya Pradesh has historically been poor, not attracting proper attention from the policy makers until 2005. In this paper we show that the development of several factors was important and made noteworthy contribution in the emergence of maternal health as a priority in Madhya Pradesh policy agenda. While grading the importance of the factors was not the purpose of the study, the emergence of political priority for maternal health in the state was, to a large extent, the result of developments taking place at national level such as the launch of the NRHM, results of various surveys, advocacy by civil society, India’s aspiration of global leadership and increased media coverage which cascaded to the state level.

Development at the state level was considered by most politicians to relate to the construction of roads, buildings and the creation of infrastructure, leaving aside overall health and maternal health in particular. However, during the last decade of the twentieth century, developments started taking place in the problem, policy and political streams at international, national and state levels, which moved towards convergence during the period 2000–05. All three streams converged to some extent in 2005, resulting in maternal health appearing prominently on the political and policy agenda in Madhya Pradesh. Key factors in the problem stream were the emerging evidence of the high magnitude of the problem, civil society’s positioning of maternal mortality as a human rights violation and increasing media coverage first at national level and later at state level. The supportive policy environment and launch of the NRHM in addition to the availability of effective policy solutions were major influencing factors in the policy stream which contributed to the emergence of maternal health on priority policy agenda in the state. The key contributing factors in the political stream were India’s aspiration of global leadership, international influence, maternal health becoming a hot topic of debate and political transition at the national and state levels. Similar to the findings of other studies in different settings [10-12,29], this study found that maternal health emerged as a political priority due to the confluence of different events and developments at international, national and state levels. In this case, the influence of the developments at international and national level permeated to the state level. We did not find any evidence that Madhya Pradesh would have taken on the issue of maternal health improvement on the policy agenda if it had not first been highlighted at the national level.

Large-scale demographic surveys such as the NFHS and the DLHS and SRS results provided credible evidence of the high magnitude of maternal mortality and the poor status of maternal health. Based on this information, civil society (beginning first at the national level) started positioning maternal mortality prominently as a human rights violation. The government of India passed the Right to Information Act in 2005 which mandated a timely response to citizens’ requests for government information. This Act helped civil society to speak out fearlessly on social issues such as the poor status of maternal health care services and give convincing evidences in favor of their arguments based on the information received from the government under this Act. The penetration of the media greatly increased in all parts of the state and the country in the early years of the twenty first century and its wider availability to the population gave more power to the media to influence policies. During this time many new electronic channels arose, increasing competition between them and also augmenting the investigative and influential role of the media which started giving more space to highlight social issues such as high maternal mortality. Increased availability and use of internet and social media also contributed in raising public awareness and drawing attentions of policy makers on the issues of poor maternal health and high MMR.

The launch of NRHM at national level in 2005 followed by the launch of it in the state contributed significantly in bringing the issues of improving maternal health and reducing maternal mortality on the priority policy agenda in Madhya Pradesh. The state government accorded high priority to various interventions for reducing the MMR of the state through concerted efforts under the NRHM. For instance, the *Janani Suraksha Yojana* (Mother Protection Scheme) has contributed to a phenomenal success in increasing the numbers of institutional deliveries. However, the rise in institutional deliveries has created a demand for synchronized efforts to ensure the availability of human resources to deal with the higher workload, the effective functioning of supply system, improved efficiency in the health system, a better health management information system and monitoring of the maternal health services. There is a need for further strengthening of the health care delivery system, especially EmOC services, and ensuring equitable availability of maternal health services through ensuring adequate availability of human resources and infrastructure. Currently, there is a supportive and conducive policy environment in the state for maternal health backed by increased political will and increased financial resources.

In 2004, the United Progressive Alliance Government recognized the importance of prioritizing social and health related issues in the policy and political agenda at the national level. This move of central government also forced other parties with governments in various states to recognize the importance of social issues and some state governments immediately started working in this direction. The *Shiv Raj Singh Chauhan* led government in Madhya Pradesh was one of the few state governments in India which prioritized maternal health and gender equity issues in their policy and political agenda. International developments have a significant influence on political agenda-setting at national and sub-national levels [30]. India’s aspirations for global leadership were raised with increased economic growth but at the same time poor health indicators and the high MMR pressurized the country to ensure the equitable distribution of benefits of higher economic growth which facilitated the translation of that information into policies and programmes for improving maternal health [31]. Developments at the international level such as the declaration of MDGs, recommendations from the World Health Organization’s Commission on Macroeconomics and Health, publications of international agencies and Jan Swasthya Abhiyan’s association with the Right to Health Campaign of the Global People’s Health Movement also influenced the processes of prioritizing maternal health at the state level [32].

The results of this study have contributed to our understanding of the complexities associated with political agenda-setting in a resource-scarce setting. Our study has highlighted that despite maternal health prominently appearing on the political agenda with a good political environment, the MMR in the state continues to be high. Malnutrition and population stabilization are the competing priorities which are receiving considerable attention from the politicians and policy makers. There are concerns among the policy actors in the state that these competing priorities may push maternal health aside from the agenda. In order to keep maternal health on the political agenda, all concerned policy actors need to be vigilant and continue playing an active role. The utilization of maternal health care is a complex phenomenon which is influenced by several socio-economic and cultural factors. The state government has taken far fewer initiatives to address these factors. Therefore, along with addressing implementation challenges associated with the current policies and programmes, the state government also needs to focus on addressing social determinants of maternal health with a human rights-based approach for improving the status of maternal health. This study also underlines that Kingdon’s multiple streams model proved to be a useful framework for analysing political priorities at sub-national level. It provided an excellent lens to assess and understand the dynamic processes related to the emergence of maternal health as a political priority in Madhya Pradesh and the influence of associated factors. This study has added to our understanding of how processes and developments at international, national and state levels in three streams interfaced and converged, resulting in the emergence of maternal health as a political priority in Madhya Pradesh.

### Methodological considerations

The study respondents were purposively selected and some important perspectives may have been omitted, e.g. one of the streams was political but we did not interview politicians. However, since qualitative research aims for theoretical generalization, our participants were selected for their ability to contribute to the research question [33]. We triangulated data by comparing the responses of study participants with findings from the literature and a review of documents. The combination of researchers, with different expertise and levels of knowledge in the area, allowed both external and internal perspectives, which also adds to the trustworthiness of the study [34]. Although there are limitations in the transferability of the study results [34], we believe that it is useful for other researchers wishing to study political priorities at the sub-national level, especially in countries with a federal structure of governance. Through the launch of the NRHM, the central government put pressure on all the states lagging behind in reducing maternal mortality including Madhya Pradesh. It would have been important to compare the processes in Madhya Pradesh with other states. However, it was not possible in this study due to the unavailability of studies on this topic in other states and logistic issues did not allow us to conduct these studies in other states. We recommend further research in this direction.

## Conclusions

The emergence of maternal health as a political priority in Madhya Pradesh in India was the result of a convergence in the developments taking place in the problem, policy and politics streams at the international, national and state levels. The factors associated with the emergence of maternal health as a political priority in Madhya Pradesh were emerging evidence of the high magnitude of maternal mortality, civil society’s positioning of maternal mortality as a human rights violation, increasing media coverage, the supportive policy environment and launch of the NRHM, availability of effective policy solutions, India’s aspiration of global leadership, international influence, maternal mortality becoming a hot topic and political transition at national and state levels. Most of these factors first became important at national level and then cascaded to the state level. The convergence of developments in the problem, policy and political streams in the year 2005 opened a window of opportunity for maternal health to become a political priority. This led to several opportunities in terms of policies, guidelines, programmes and investments for improving maternal health. These efforts have been successful to some extent but there are several challenges in terms of implementation which require special attention. Our study has also highlighted the need to focus on addressing social determinants of maternal health with a human rights-based approach along with improving the maternal health service delivery in the state. Decision makers and advocacy groups active at the sub-national level in other settings can gain insights on how maternal health became a political priority at the state level as a result of the convergence of streams and the interface of processes at different levels.

## Competing interests

The authors declare that they have no competing interests.

## Authors’ contributions

TRJ and MSS conceptualized and designed the study. TRJ performed data collection, analyzed the data and prepared the first draft of the manuscript. All the authors interpreted the results and worked for revising various drafts of the manuscript. All the authors have read and approved the final manuscript.

## Pre-publication history

The pre-publication history for this paper can be accessed here:

http://www.biomedcentral.com/1471-2393/13/181/prepub
